# Reducing Periocular Edema: Review and Product Concept

**DOI:** 10.7759/cureus.77815

**Published:** 2025-01-22

**Authors:** Isha Gandhi, Robert Adler, Chase Fishman, Fariha Khan, Mark Albert

**Affiliations:** 1 Dermatology, University of Minnesota Twin Cities Medical School, Minneapolis, USA; 2 Dermatology, State University of New York (SUNY) Downstate Medical School, Brooklyn, USA; 3 Sports Medicine, Massachusetts Institute of Technology, Cambridge, USA; 4 Dermatology, Touro College of Osteopathic Medicine, New York City, USA; 5 Plastic Surgery, Albert Plastic Surgery, New York City, USA

**Keywords:** edema reduction, enswell, mod-enswell, periocular edema, periorbital edema

## Abstract

Prompt and gentle reduction of periocular edema is imperative. Here, we comprehensively review diverse accepted and novel strategies to mitigate periocular edema including corticosteroids, nonsteroidal anti-inflammatory drugs (NSAIDs), bromelain, diuretics, surgical and other non-pharmaceutical methods, and cryotherapy. We also introduce the concept for an innovative cryotherapeutic device: Mod-Enswell. Made of surgical steel, Mod-Enswell consists of a rectangular base with short pegs extending from its surface. The device was designed to induce focal vasoconstriction and gradually cool skin, features that are especially important considering the delicate nature of periocular skin. This paper explores various avenues to improve patient recovery following periocular swelling; future comparative investigations will be needed to determine the ideal strategy to reduce periocular edema.

## Introduction and background

Periocular trauma is a common reason for emergency room visits, with causes ranging from sports injuries and motor vehicle accidents to fireworks [1-3]. Between 2006 and 2011, ocular trauma represented 77.9% of ophthalmic presentations in emergency rooms nationally [4]. 

The periocular area, with skin that can be as thin as 0.2 mm, is very susceptible to inflammation and injury [4,5]. Periocular edema can have several etiologies ranging from blunt force trauma to endocrinological, immunological, infectious disease, neurological, and dermatological conditions. Sometimes, periocular edema is the sole symptom of a systemic disease, such as lupus, prior to its diagnosis [6,7]. Initial presentation of periocular edema includes symptoms such as erythema, chemosis and a fatigued appearance. 

In the postoperative setting, periocular edema can present either as a result of the procedure, as an exacerbation of a previously associated condition, or due to medications during the procedure. Conditions such as cold urticaria and eyelid-dependent keratoconjunctivitis sicca are common iatrogenic causes of ocular edema [8]. General anesthesia and lidocaine have also been reported to cause periocular edema [9,10]. While post-procedural swelling can vary, edema will generally increase for 48 hours and maximize at 72 hours [5].

However, persistent periocular edema can spread to the conjunctiva, leading to progression of symptoms such as chemosis with corneal desiccation, thinning, and ulceration paired with significant visual disturbances [8]. Moreover, even small alterations in the periocular area can result in dramatic physical appearance changes. For instance, tear trough deformities can result in a sunken eye appearance, creating dark shadows and causing a fatigued appearance despite adequate rest [11]. 

Considering the adverse clinical outcomes associated with prolonged edema and the nature of inflammation, prompt recovery is imperative [12]. Here, we provide an overview of strategies for minimizing postoperative periocular edema and introduce the concept for a novel cryotherapy device, Mod-Enswell, for this purpose. 

## Review

Given the limited availability of studies focusing specifically on reducing periocular edema, our literature search was expanded to include relevant publications from 2000 to 2023. PubMed and Google Scholar were utilized to identify pertinent literature related to perioperative periocular edema. The search strategy incorporated a range of relevant keywords, including but not limited to 'postoperative edema,' 'periorbital edema,' and 'edema' to ensure comprehensive coverage of the topic. Literature selection was based on relevance to the study objectives, with a particular focus on studies addressing the management of perioperative periocular edema. Preference was given to peer-reviewed articles.

Pathogenesis of perioperative perioclular edema

Approximately two-thirds of the body’s fluid is contained within cells; the remaining third is in the extracellular compartment. Within the extracellular space, 60% resides in the interstitial fluid, with the remaining 40% in the intravascular space [13,14]. Edema occurs when there is accumulation of excess fluid within the interstitial compartment of tissues or organs [13].

Movement of fluid into the interstitium can arise from increased interstitial oncotic pressure, due to large molecules such as albumin, or from elevated hydrostatic pressure within the intravascular compartment. In a healthy physiological state, hydrostatic pressure marginally surpasses extracellular oncotic pressure, leading to a net movement of fluid into the interstitium. This fluid is subsequently collected by the lymphatic system and returned to venous circulation [13].

**Figure 1:**
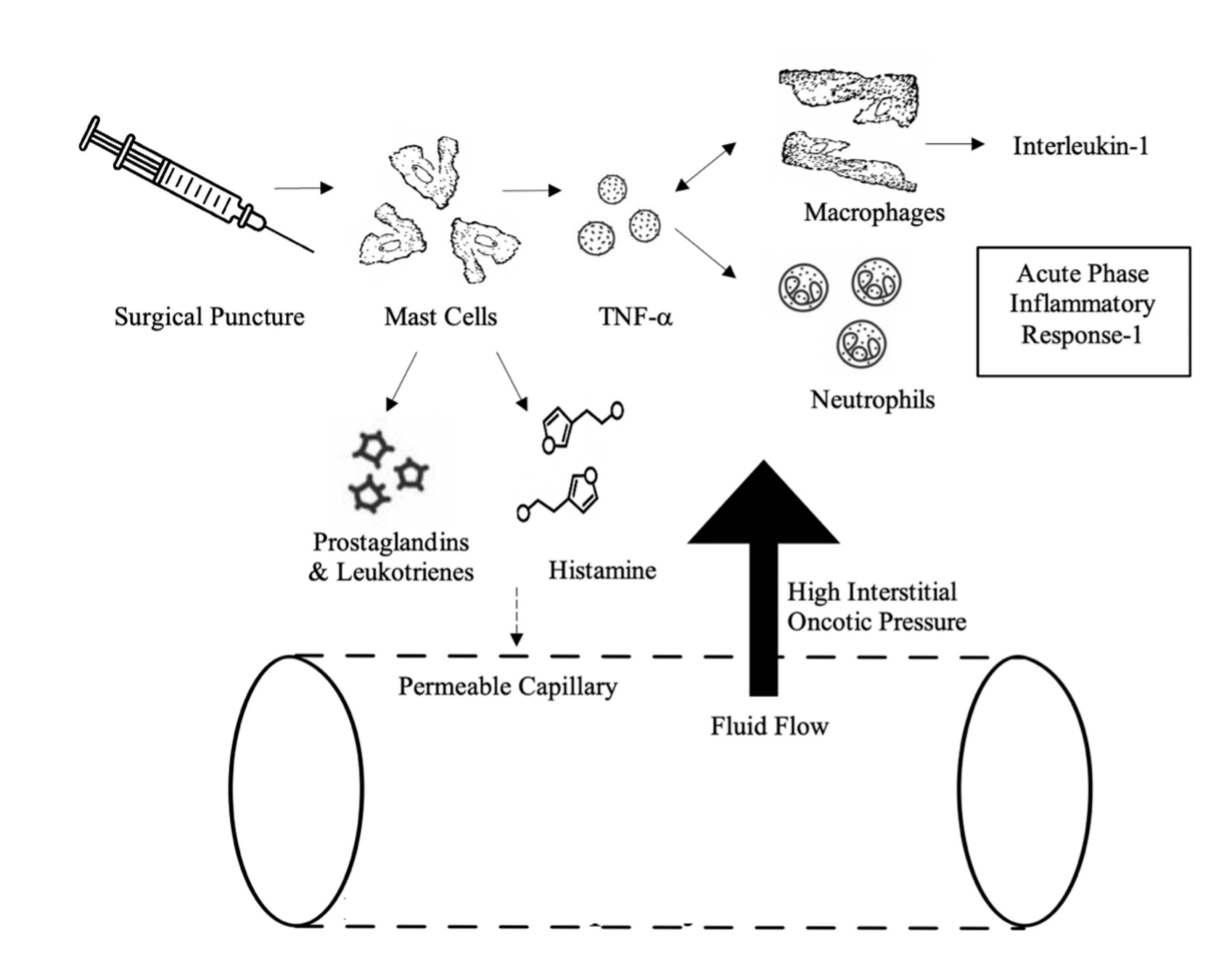
Simplified Schematic of Interstitial Edema Surgical puncture triggers mast cells to release histamine, prostaglandin, and leukotrienes which increase vascular permeability. Mast cells, amongst other immune cells, also secrete tumor necrosis factor (TNF) which attracts macrophages to injury sites. Macrophages subsequently release interleukin-1 (IL-1) and additional TNF, attracting neutrophils and stimulating the acute phase inflammatory response. Prostaglandins and leukotrienes are released during this acute phase response, enhancing the permeability of the capillary membrane. The influx of immune cells and inflammatory cytokines increases interstitial oncotic pressure. This combination of heightened vascular permeability and increased interstitial oncotic pressure draws fluid into the interstitium, leading to edema. Image credits: Isha Gandhi

In response to surgical incision or puncture, the immune system releases cytokines to promote tissue repair, minimize injury, and prevent infection. Namely, mast cells release histamine and prostaglandin, inducing local vasodilation and increased vascular permeability. Mast cells, amongst other immune cells, also secrete tumor necrosis factor (TNF) which attracts macrophages to injury sites [15,16]. Macrophages subsequently release interleukin-1 (IL-1) and additional TNF, attracting neutrophils and stimulating the acute phase inflammatory response [17,18]. Prostaglandins and leukotrienes are released during this acute phase response, enhancing the permeability of the capillary membrane. The influx of immune cells and inflammatory cytokines increases interstitial oncotic pressure. This combination of heightened vascular permeability and increased interstitial oncotic pressure draws fluid into the interstitium, leading to edema (Figure 1). 

In the periocular area, where the skin is very thin, this excess fluid stretches the tissues and can lead to a prominent edematous appearance. Moreover, the periocular area has a rich network of lymphatics. During states of inflammation, lymphatic drainage can be impaired, prolonging the presence of edema. Rapidly resolving periocular swelling without compromising the delicate surrounding skin is vital towards ensuring optimal healing and appearance. 

Methods to reduce periocular swelling

Prevention of Periocular Edema

While management of periocular swelling in clinic can be accomplished, prevention is ideal. Occupation-related ocular injury presents a significant public health risk, with agricultural and construction workers at higher risk of developing ocular-related injury. Encouraging the use of safety goggles amongst workers most at risk is one of the foremost prevention methods, with studies demonstrating peer encouragement a significant driver of increased use of protective wear and reduction in ocular injury [19,20]. 

Corticosteroids

Topically applied: Topical corticosteroids are widely used, alleviating various conditions from dermatitis to alopecia areata. Their efficacy and side effects depend on their anti-inflammatory and immunosuppressive properties [21]. These topical treatments are classified by their potency, a measure of their vasoconstrictive properties, and can be considered low, medium, or high potency. 

Betamethasone valerate (BMV), a medium potency corticosteroid without mineralocorticoid properties, binds to glucocorticoid receptors and subsequently DNA. This interaction results in the synthesis of anti-inflammatory proteins and inhibits the synthesis of certain inflammatory mediators [22]. Due to its anti-inflammatory properties and non-invasive application, BMV can be used to treat cutaneous periocular edema. A study conducted in 2013 emphasized the efficacy of BMV plasters in alleviating facial edema and inflammation. The study further demonstrated that patients were significantly more satisfied with a betamethasone plaster to reduce facial edema when compared to intradermally administered Acesin cream [23].

Triamcinolone, also a medium-potency corticosteroid, has comparable efficacy to betamethasone dipropionate - more potent than BMV - for the treatment of several conditions [24,25]. Yet, a study demonstrated that achieving the same therapeutic effect with betamethasone dipropionate necessitates a concentration 4.6 times greater than triamcinolone [26]. Given the delicate nature of the periocular skin, treating edema with the less potent BMV may be more beneficial than using triamcinolone in this region.

However, judicious use of BMV is imperative when treating the periocular skin due to its delicate nature. Prolonged use of topical steroids can result in decreased skin elasticity and epidermal thinning [27]. Due to the immunosuppressive and anti-inflammatory properties of topical corticosteroids, prolonged use may result in other side effects: cutaneous infections, rosacea, acne, hypopigmentation, among others. Resolution of these side effects necessitates discontinuing topical steroids, but remission can take months [21].

Systemic steroids: Perioperative corticosteroids can reduce short-term periocular edema by mitigating vascular permeability and providing anti-inflammatory effects [27].

The efficacy of dexamethasone in significantly reducing periocular edema in both the upper and lower eyelids, in comparison to placebo, has been established [5,28]. A 2018 study demonstrated that a minimal intravenous dose of dexamethasone (8 mg) can significantly alleviate periocular edema without triggering any adverse effects in rhinoplasty patients [29]. 

Prednisone, a glucocorticoid with a shorter half-life than dexamethasone, has also been utilized to mitigate periocular edema [5]. Although limited literature exists comparing dexamethasone and prednisone for edema reduction, trials evaluating both for asthma favored dexamethasone due to its faster action and comparatively fewer side effects [30,31].

The systemic immunosuppressive and endocrine properties of oral corticosteroids can cause several adverse effects. Short-term oral steroid use has been associated with increased intraoperative bleeding and avascular necrosis [27]. Long-term steroid use can lead to a spectrum of symptoms encompassing fatigue, psychiatric disturbances, gastrointestinal symptoms (nausea, vomiting, abdominal pain), dermatological issues (acne, facial erythema, perioral dermatitis), and an increased susceptibility to infection [32].

Given the potential side effects of steroids, first-line treatment for prolonged procedure-induced edema, such as hyaluronic acid for tear trough fillers, typically involves nonsteroidal anti-inflammatory drugs (NSAIDs) [33]. However, if persistent swelling or allergic reactions occur, systemic corticosteroids and antihistamines are recommended [34-36].

Non-steroidal Anti-inflammatory Drugs

NSAIDs are part of the first-line treatment recommendations for prolonged periocular edema following procedures [33]. NSAIDs inhibit cyclooxygenase one and two, curtailing the synthesis of prostaglandins and subsequently reducing vascular permeability and edema [37]. Notably, prior to many in-office procedures, NSAIDs should be avoided for at least five days to prevent bruising [11]. Moreover, NSAIDs are contraindicated in patients with gastrointestinal issues such as peptic ulcers, renal impairment, and NSAID hypersensitivity [37]. 

Bromelain

Bromelain, a protease derived from pineapples, has potential as a therapeutic agent to reduce periocular edema in the post-operative setting. It has anti-inflammatory, anti-edematous, and antithrombotic effects. A substantial amount of bromelain can be absorbed without losing its proteolytic activity or causing adverse effects [38]. Mechanistically, bromelain reduces inflammation by inhibiting bradykinin production, suppressing leukocyte migration by CD128 receptor antagonism, and enhancing IL-2 which modulates the inflammatory response through Treg activation [39]. Clinically, bromelain has significantly reduced periocular edema in rhinoplasty patients [40]. However, there have been studies that report mixed results on the efficacy of bromelain in reduction of edema, necessitating further research [41]. Notably, bromelain is contraindicated in children due to lack of clinical safety studies [42]. Thus, judicious use of bromelain for periocular edema is imperative. 

Surgical and Post-procedural Techniques

Plastic surgeons have explored numerous interventions for periocular edema reduction post-rhinoplasty. A common technique is nasal packing; studies both refute and support the efficacy of this concept in reducing edema post-rhinoplasty. Sowerby et al. found decreased swelling and periocular edema post nasal packing, whereas Arfaj found increased swelling [43,44].

An additional method is periosteal elevation, by which a subperiosteal tunnel is created during lateral osteotomy rhinoplasty; however this method has also produced varied results, with studies showing both increases and decreases in post-operative periocular edema [45,46]. A promising technique that has been studied is the combination of corticosteroids with cold saline gauze compression, which demonstrated decreased edema compared to controls [47]. Additional data is needed to determine the effect of cold saline gauze independent of corticosteroid therapy. 

Many practices for reducing periocular edema in plastic surgery settings, such as cryotherapy following blepharoplasty, have potential and may be applied towards other periocular edema etiologies.

Cryotherapy

Cryotherapy is well established in mitigating edema, bruising and erythema [48]. This approach induces vasoconstriction and reduces recruitment of leukocytes, such as macrophages and neutrophils [48]. By disrupting the inflammatory cascade, cryotherapy can alleviate pain and edema [49,50].

However, excessive cooling reduces blood flow, thereby compromising wound healing and extending recovery time [51]. In evaluating whole-body cryotherapy in athletes, serum studies discovered decreased levels of IL-18 and IL2RA, but also decreased ferritin, mean corpuscular hemoglobin, and mean platelet volume, which while within normal range can increase the risk of compromised wound healing [52]. Given the thin nature of periocular skin, avoiding overcooling in this area is imperative. Ice packs, often too cold for the delicate periocular skin, can exacerbate this concern. This is especially important as periocular presentations are common and require swift recovery [5,53]. Therefore, choosing a cryotherapeutic modality that capitalizes on skin-cooling advantages while avoiding its adverse side effects is imperative. Mod-Enswell is a novel device that addresses this necessary balance. 

Mod-Enswell

**Figure 2:**
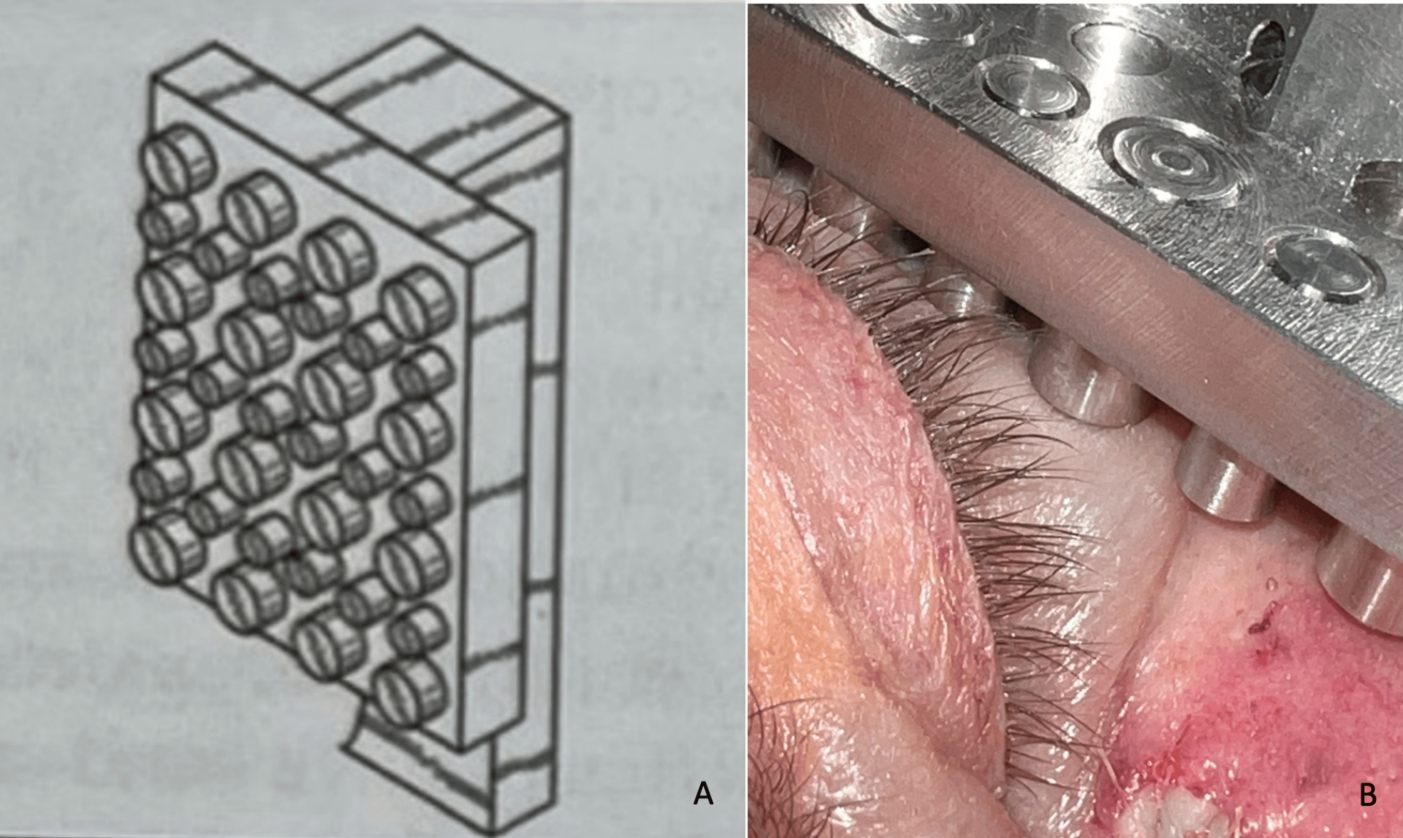
Mod-Enswell Device Panel A depicts a schematic model of Mod-Enswell. Mod-Enswell is designed with a surgical steel rectangular base with multiple short steel pegs extending from its surface. Panel B depicts Mod-Enswell being used on a patient. Image credits: Chase Fishman

Mod-Enswell is designed with a surgical steel rectangular base with multiple short steel pegs extending from its surface (Figure 2). A 2020 study demonstrated that surgical steel produces significantly less skin temperature cooling within 60 seconds compared to commercial ice packs [54]. This characteristic allows Mod-Enswell to leverage the benefits of skin cooling while circumventing its adverse side effects, rendering it a promising cryotherapeutic candidate for mitigating edema in the delicate periocular area. 

Moreover, the undulating steel pegged surface of Mod-Enswell distributes pressure, thereby preventing the blunt pressure-induced damage that can be associated with traditional ice packs. This distributed pressure also reduces the risk of hematoma and clotting. Additionally, the pegged surface facilitates focal vasoconstriction, thus preserving the wound-healing benefits of blood flow while mitigating the inflammatory cascade [55]. Contraindications include allergy to steel and active, profuse bleeding. Adverse effects (AEs) may include discomfort and freezing cold injury. However, the risk of freezing cold injury with this device is lower compared to ice, as steel results in significantly less skin temperature reduction within 60 seconds compared to commercial ice packs [54].

Given Mod-Enswell’s potential to curtail hematoma, reduce edema, and expedite recovery, it is an ideal candidate for clinical evaluation in patients in the perioperative as well as those initially presenting with edema. This is especially significant given the minimally invasive nature of such interventions. 

## Conclusions

Numerous modalities have been employed to mitigate edema in medical management and procedural settings. While conventional methods such as topical corticosteroids, systemic steroids, and ice packs offer benefits, innovative options such as bromelain and Mod-Enswell have the potential to effectively address edema while reducing undesirable effects. Despite their therapeutic potential, these innovative options have not been clinically evaluated for efficacy in management of periocular edema. Comprehensive comparative investigations are necessary to ascertain the optimal approach to reduce periocular edema in this context.

## References

[1] McLeod G, O'Connor S, Morgan D, Kountouris A, Finch CF, Fortington LV (2020). Medical-attention injuries in community cricket: a systematic review. BMJ Open Sport Exerc Med.

[2] Cade KL, Taneja K, Jensen A, Rajaii F (2023). Incidence, characteristics, and cost of eyelid lacerations in the United States from 2006 to 2014. Ophthalmol Ther.

[3] Lenglinger MA, Zorn M, Pilger D, von Sonnleithner C, Rossel M, Salchow DJ, Bertelmann E (2021). Firework-inflicted ocular trauma in children and adults in an urban German setting. Eur J Ophthalmol.

[4] Haring RS, Canner JK, Haider AH, Schneider EB (2016). Ocular injury in the United States: emergency department visits from 2006-2011. Injury.

[5] Niamtu J 3rd (2004). Cosmetic blepharoplasty. Atlas Oral Maxillofac Surg Clin North Am.

[6] Sliman RK, Saied MH (2022). Periorbital edema as the initial manifestation of pediatric systemic lupus erythematosus. Clin Med Insights Case Rep.

[7] Erras S, Benjilali L, Essaadouni L (2012). Periorbital edema as initial manifestation of chronic cutaneous lupus erythematosus. Pan Afr Med J.

[8] Sami MS, Soparkar CN, Patrinely JR, Tower RN (2007). Eyelid edema. Semin Plast Surg.

[9] Presman B, Vindigni V, Tocco-Tussardi I (2016). Immediate reaction to lidocaine with periorbital edema during upper blepharoplasty. Int J Surg Case Rep.

[10] Tobias JD, Jagannathan N, Sawardekar A, Bhalla T (2011). An unusual cause of post-operative orbital edema in a child after general anesthesia. Saudi J Anaesth.

[11] Sharad J (2012). Dermal fillers for the treatment of tear trough deformity: a review of anatomy, treatment techniques, and their outcomes. J Cutan Aesthet Surg.

[12] Vaughan-Shaw PG, Saunders J, Smith T, King AT, Stroud MA (2013). Oedema is associated with clinical outcome following emergency abdominal surgery. Ann R Coll Surg Engl.

[13] van Wissen K, Breton C (2004). Perioperative influences on fluid distribution. Medsurg Nurs.

[14] Lent-Schochet D, Jialal I (2023). Physiology, edema. StatPearls.

[15] Gu Y, Yang DK, Spinas E (2015). Role of TNF in mast cell neuroinflammation and pain. J Biol Regul Homeost Agents.

[16] Mosser DM, Edwards JP (2008). Exploring the full spectrum of macrophage activation. Nat Rev Immunol.

[17] Laskin DL, Sunil VR, Gardner CR, Laskin JD (2011). Macrophages and tissue injury: agents of defense or destruction?. Annu Rev Pharmacol Toxicol.

[18] Ott LW, Resing KA, Sizemore AW (2007). Tumor necrosis factor-alpha- and interleukin-1-induced cellular responses: coupling proteomic and genomic information. J Proteome Res.

[19] Monaghan PF, Forst LS, Tovar-Aguilar JA (2011). Preventing eye injuries among citrus harvesters: the community health worker model. Am J Public Health.

[20] Chatterjee S, Agrawal D (2017). Primary prevention of ocular injury in agricultural workers with safety eyewear. Indian J Ophthalmol.

[21] Ference JD, Last AR (2009). Choosing topical corticosteroids. Am Fam Physician.

[22] Chapman A (1977). [Preventive treatment of acute renal failure in cardiac surgery]. Ann Anesthesiol Fr.

[23] Iannitti T, Rottigni V, Palmieri B (2013). Corticosteroid transdermal delivery to target swelling, edema and inflammation following facial rejuvenation procedures. Drug Des Devel Ther.

[24] Saki N, Hosseinpoor S, Heiran A, Mohammadi A, Zeraatpishe M (2018). Comparing the efficacy of triamcinolone acetonide iontophoresis versus topical calcipotriol/betamethasone dipropionate in treating nail psoriasis: a bilateral controlled clinical trial. Dermatol Res Pract.

[25] de Sousa VB, Arcanjo FP, Aguiar F, Vasconcelos J, Oliveira AF, Honório A, Pontes J (2022). Intralesional betamethasone versus triamcinolone acetonide in the treatment of localized alopecia areata: a within-patient randomized controlled trial. J Dermatolog Treat.

[26] Coondoo A, Phiske M, Verma S, Lahiri K (2014). Side-effects of topical steroids: a long overdue revisit. Indian Dermatol Online J.

[27] Coroneos CJ, Voineskos SH, Cook DJ, Farrokyar F, Thoma A (2016). Perioperative corticosteroids reduce short-term edema and ecchymosis in rhinoplasty: a meta-analysis. Aesthet Surg J.

[28] Aldhabaan SA, Hudise JY, Obeid AA (2022). A meta-analysis of pre- and postoperative corticosteroids for reducing the complications following facial reconstructive and aesthetic surgery. Braz J Otorhinolaryngol.

[29] Sanober A, Rashid M, Khan MI (2018). Use of steroids in rhinoplasty with lateral osteotomies for reducing post operative oedema. JAMC.

[30] Shefrin AE, Goldman RD (2009). Use of dexamethasone and prednisone in acute asthma exacerbations in pediatric patients. Can Fam Physician.

[31] Banoth B, Verma A, Bhalla K, Khanna A, Holla S, Yadav S (2022). Comparative effectiveness of oral dexamethasone vs. oral prednisolone for acute exacerbation of asthma: a randomized control trial. J Family Med Prim Care.

[32] Yasir M, Goyal A, Sonthalia S (2023). Corticosteroid adverse effects. StatPearls.

[33] Guduk SS (2018). An unusual delayed type reaction following periorbital filler injection with hyaluronic acid. Aesthet Surg J.

[34] Lafaille P, Benedetto A (2010). Fillers: contraindications, side effects and precautions. J Cutan Aesthet Surg.

[35] Van Dyke S, Hays GP, Caglia AE, Caglia M (2010). Severe acute local reactions to a hyaluronic acid-derived dermal filler. J Clin Aesthet Dermatol.

[36] Funt DK (2022). Treatment of delayed-onset inflammatory reactions to hyaluronic acid filler: an algorithmic approach. Plast Reconstr Surg Glob Open.

[37] Frishman WH (2002). Effects of nonsteroidal anti-inflammatory drug therapy on blood pressure and peripheral edema. Am J Cardiol.

[38] Agrawal P, Nikhade P, Patel A, Mankar N, Sedani S (2022). Bromelain: a potent phytomedicine. Cureus.

[39] Ordesi P, Pisoni L, Nannei P, Macchi M, Borloni R, Siervo S (2014). Therapeutic efficacy of bromelain in impacted third molar surgery: a randomized controlled clinical study. Quintessence Int.

[40] Sakallioglu O, Gülmez E, Yildirim YSS, Cetiner H, Duzer S, Susaman N (2021). Effect of bromelain and arnica combination on periorbital edema and ecchymosis in septorhinoplasty [Preprint]. Authorea.

[41] Ho D, Jagdeo J, Waldorf HA (2016). Is there a role for arnica and bromelain in prevention of post-procedure ecchymosis or edema? A systematic review of the literature. Dermatol Surg.

[42] (2023). Bromelain Information. https://www.mountsinai.org/health-library/supplement/bromelain.

[43] Al Arfaj AM (2015). The use of nasal packing post rhinoplasty: does it increase periorbital ecchymosis? A prospective study. J Otolaryngol Head Neck Surg.

[44] Sowerby L, Kim LM, Chow W, Moore C (2019). Intra-operative nasal compression after lateral osteotomy to minimize post-operative peri-orbital ecchymosis and edema. J Otolaryngol Head Neck Surg.

[45] Kara CO, Kara IG, Topuz B (2005). Does creating a subperiosteal tunnel influence the periorbital edema and ecchymosis in rhinoplasty?. J Oral Maxillofac Surg.

[46] Al-Arfaj A, Al-Qattan M, Al-Harethy S, Al-Zahrani K (2009). Effect of periosteum elevation on periorbital ecchymosis in rhinoplasty. J Plast Reconstr Aesthet Surg.

[47] Taskin U, Yigit O, Bilici S, Kuvat SV, Sisman AS, Celebi S (2011). Efficacy of the combination of intraoperative cold saline-soaked gauze compression and corticosteroids on rhinoplasty morbidity. Otolaryngol Head Neck Surg.

[48] Rohrich RJ, Bartlett EL, Dayan E (2019). Practical approach and safety of hyaluronic acid fillers. Plast Reconstr Surg Glob Open.

[49] Schaser KD, Disch AC, Stover JF, Lauffer A, Bail HJ, Mittlmeier T (2007). Prolonged superficial local cryotherapy attenuates microcirculatory impairment, regional inflammation, and muscle necrosis after closed soft tissue injury in rats. Am J Sports Med.

[50] Sloan JP, Giddings P, Hain R (1988). Effects of cold and compression on edema. Phys Sportsmed.

[51] Collins NC (2008). Is ice right? Does cryotherapy improve outcome for acute soft tissue injury?. Emerg Med J.

[52] Selleri V, Mattioli M, Lo Tartaro D (2022). Innate immunity changes in soccer players after whole-body cryotherapy. BMC Sports Sci Med Rehabil.

[53] Liew S, Nguyen DQ (2011). Nonsurgical volumetric upper periorbital rejuvenation: a plastic surgeon's perspective. Aesthetic Plast Surg.

[54] Beauregard TA, Vaile J, Whitney L, Merrick M, Moody V (2020). Rapid facial cryotherapy for combat sports: a comparison of materials and methods for cooling facial tissues in 60 Seconds or less. J Sport Rehabil.

[55] Khan F, Fishman C, Albert M (2022). An innovative tool to treat post-procedural periocular swelling. Cutis.

